# Time to abandon ampicillin plus gentamicin in favour of ampicillin plus ceftriaxone in *Enterococcus faecalis* infective endocarditis? A meta-analysis of comparative trials

**DOI:** 10.1007/s00392-021-01971-3

**Published:** 2021-11-09

**Authors:** Moritz Mirna, Albert Topf, Lukas Schmutzler, Uta C. Hoppe, Michael Lichtenauer

**Affiliations:** Division of Cardiology, Department of Internal Medicine II, Paracelsus Medical University of Salzburg, Müllner Hauptstraße 48, 5020 Salzburg, Austria

**Keywords:** Cardiology, Endocarditis, Meta-analysis, Enterococcus, Ampicillin, Ceftriaxone, Gentamicin

## Abstract

**Background:**

Current guidelines recommend either ampicillin plus ceftriaxone (AC) or amoxicillin/ampicillin plus gentamicin (AG) with an equivalent class IB recommendation in *Enterococcus faecalis* endocarditis. However, previous observational studies suggest that AC might be favourable in terms of adverse events.

**Objectives:**

To investigate whether AC is non-inferior to AG, and if it is associated with less adverse events.

**Methods:**

In June 2021, a systematic literature search using the databases PubMed/MEDLINE, CDSR, CENTRAL, CCAs, EBM Reviews, Web of Science and LILACS was conducted by two independent reviewers. Studies were considered eligible if (P) patients included were ≥ 18 years of age and had IE with *E. faecalis*, (I) treatment with AC was compared to (C) treatment with AG and (O) outcomes on in-hospital mortality, nephrotoxicity and adverse events requiring drug withdrawal were reported. Odds ratios and 95% confidence intervals were calculated using random-effects models with the Mantel–Haenszel method, the Sidik–Jonkman estimator for *τ*^*2*^ and the Hartung–Knapp adjustment.

**Results:**

Treatment with AC was non-inferior to AG, as depicted by no significant differences in-hospital mortality, 3-month mortality, relapses or treatment failure. Furthermore, AC was associated with a lower prevalence of nephrotoxicity (OR 0.45 [0.26–0.77], *p* = 0.0182) and drug withdrawal due to adverse events (OR 0.11 [0.03–0.46], *p* = 0.0160) than AG.

**Conclusions:**

Treatment with AC was non-inferior to treatment with AG, and it was associated with a reduced prevalence of nephrotoxicity and drug withdrawal due to adverse events. Thus, combination therapy with AC appears favourable over AG in patients with *E. faecalis* IE.

**Graphical abstract:**

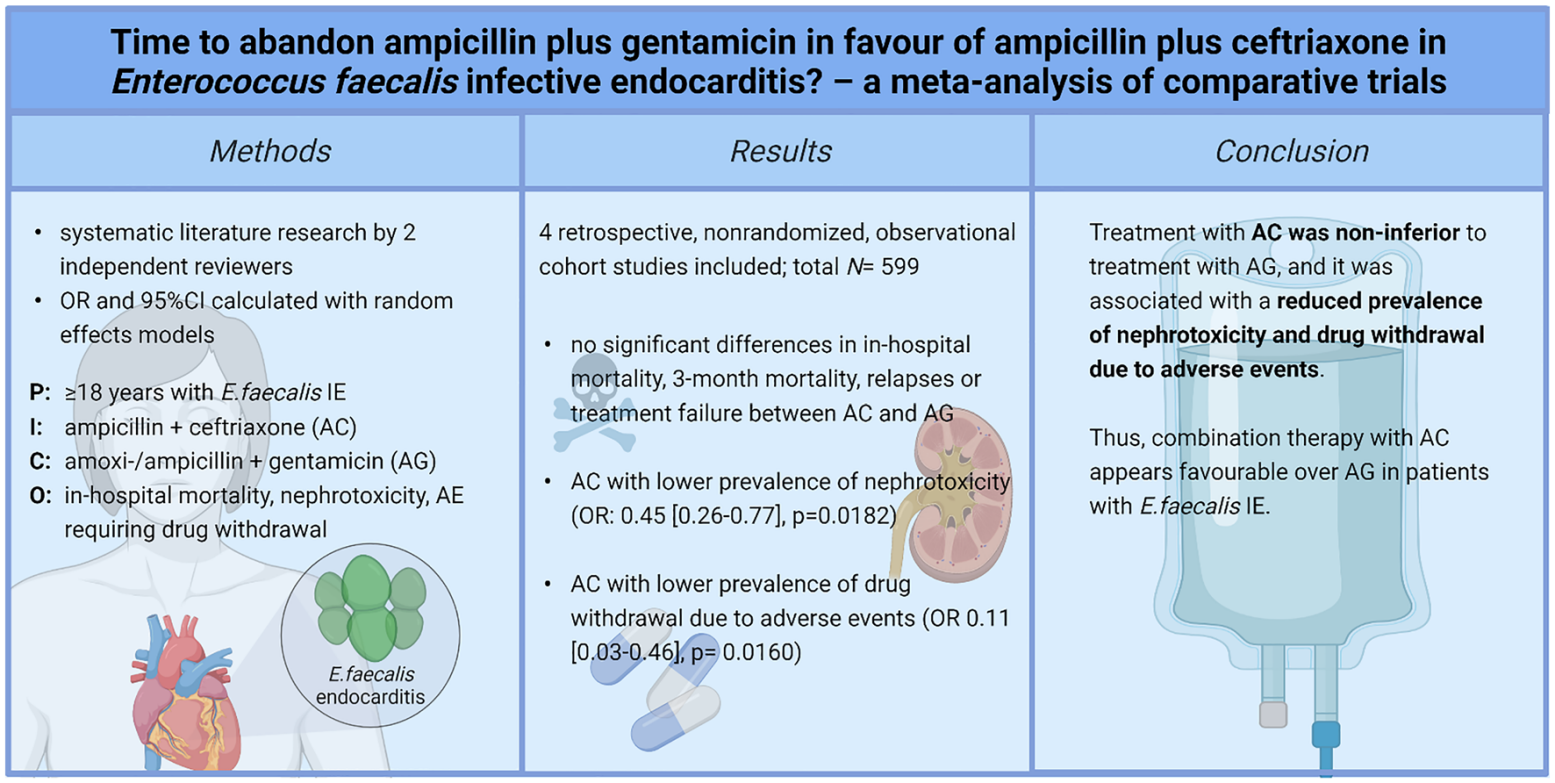

**Supplementary Information:**

The online version contains supplementary material available at 10.1007/s00392-021-01971-3.

## Introduction

Due to its elaborated and tedious treatment regimens, as well as its associated morbidity and mortality, infective endocarditis (IE) frequently poses a challenge to the attending physician in clinical practice. Endocarditis can be caused by various different microorganisms, with *Enterococci* spp. representing the third most common group of pathogens, with an estimated frequency of 10% of all cases [1]. Among all cases of enterococcal IE, *Enterococcus faecalis* further represents the predominant strain of *Enterococci*, with a prevalence of almost 90% [2].

To date, the European Society of Cardiology (ESC) recommends both a combination of two cell wall inhibitors, namely ampicillin plus ceftriaxone for 6 weeks (AC), or one cell wall inhibitor with an aminoglycoside antibiotic, i.e., amoxicillin/ampicillin plus gentamicin for 2–6 weeks (AG), with equivalent class IB recommendations for the treatment of *E. faecalis* IE [3]. However, alarmingly high rates of high-level aminoglycoside-resistant strains of *E. faecalis* and *E. faecium* (HLAR; aminoglycoside Minimal Inhibitory Concentration (MIC) > 500 mg/L) have been reported in previous studies [2, 4, 5], which may preclude the proclaimed synergistic bactericidal effects of a combination of cell wall inhibitor plus aminoglycoside [6].

Furthermore, treatment with aminoglycosides is associated with an increased risk for adverse effects, such as drug-induced nephrotoxicity or ototoxicity [7–9], and the dosing and monitoring of these antibiotics can be quite challenging in clinical practice, especially in patients with already impaired kidney function, elderly or obese patients [10, 11].

Previous observational studies reported that combination therapy with AC could be non-inferior to treatment with AG in patients with *E. faecalis* IE in terms of mortality and relapses, as well as beneficial regarding the risk for drug-induced adverse events [12–14]. However, there is currently no high-level evidence available to support this notion. Therefore, we aimed to provide a comprehensive meta-analysis on all comparative studies regarding combination therapy of AC vs. AG in *E. faecalis* IE to further support therapeutic decisions in affected patients.

## Methods

The study was conducted according to the current PRISMA guidelines [15], the principles of Good Clinical Practice and the Declaration of Helsinki. Prior to literature research, the study protocol was registered at the international prospective register of systematic reviews PROSPERO (ID: CRD42021268601) [16].

### Data sources and search strategy

Literature search was performed by two independent reviewers (MM and LS) using predefined search terms (‘endocarditis’, ‘aminoglycoside’, ‘gentamicin’, ‘beta lactam’, ‘cephalosporin’, ‘ceftriaxone’, ‘penicillin’, ‘ampicillin’, ‘amoxicillin’, ‘enterococcus’ and ‘enterococ*’) in the databases PubMed/MEDLINE, Cochrane Database of Systematic Reviews (CDSR), Cochrane Central Register of Controlled Trials (CENTRAL), Cochrane Clinical Answers (CCAs), EBM Reviews—Database of Abstracts of Reviews of Effects, Web of Science and Latin American and Caribbean Health Sciences Literature (LILACS) in June 2021. Additionally, manual search was performed by screening all references of eligible studies.

### Study selection

Studies were considered eligible if (P) patients included were ≥ 18 years of age and had infective endocarditis with *Enterococcus faecalis*, (I) treatment with ampicillin–ceftriaxone (AC) was compared to (C) ampicillin–gentamicin (AG) and (O) outcomes on in-hospital mortality, nephrotoxicity and adverse events requiring drug withdrawal were reported. Exclusion criteria were: studies including patients < 18 years of age, studies on prophylactic treatment, animal studies or in vitro studies, studies on blood culture negative infective endocarditis (BCIEN), studies where data could not reliably be extracted, overlapping data, abstract-only papers, conference papers, editorials, books, articles without available full text, case reports and case series, as well as systematic reviews. No limits were set regarding the language and publication date of studies. Infective endocarditis was defined by the modified Duke criteria [17].

### Outcomes

The primary outcome was in-hospital mortality. Secondary outcome measures were 3-month mortality, nephrotoxicity, adverse effects requiring drug withdrawal, relapse within 3 months of follow-up (defined as isolation of *E. faecalis* in blood specimens within the follow-up period) and treatment failure requiring change of antibiotic regimen.

### Statistical analyses

Statistical analyses were performed with R (version 4.0.2., R Core Team (2013), R Foundation for Statistical Computing, Vienna, Austria; http://www.R-project.org/) using the packages ‘Rcmdr’, ‘ggplot2’, ‘meta’, ‘metafor’, ‘dplyr’ and ‘dmetar’. Odds ratios and 95% confidence intervals (CI) were calculated using random-effects models with the Mantel–Haenszel method, the Sidik–Jonkman estimator for *τ*^*2*^ and the Hartung–Knapp adjustment. Continuity correction was performed with treatment arm continuity correction (TACC). Between-study heterogeneity was assessed by calculating *τ*^*2*^, Higgin’s &Thompson’s *I*^*2*^*,* Cochran’s *Q*, prediction intervals and by Baujat plot. Risk of bias assessment was conducted using the rob.summary() function of the ‘dmetar’ package for R, which is based on the Cochrance Risk of Bias Tool. A *p* value < 0.05 was considered statistically significant.

## Results

### Selected studies

**Fig. 1:**
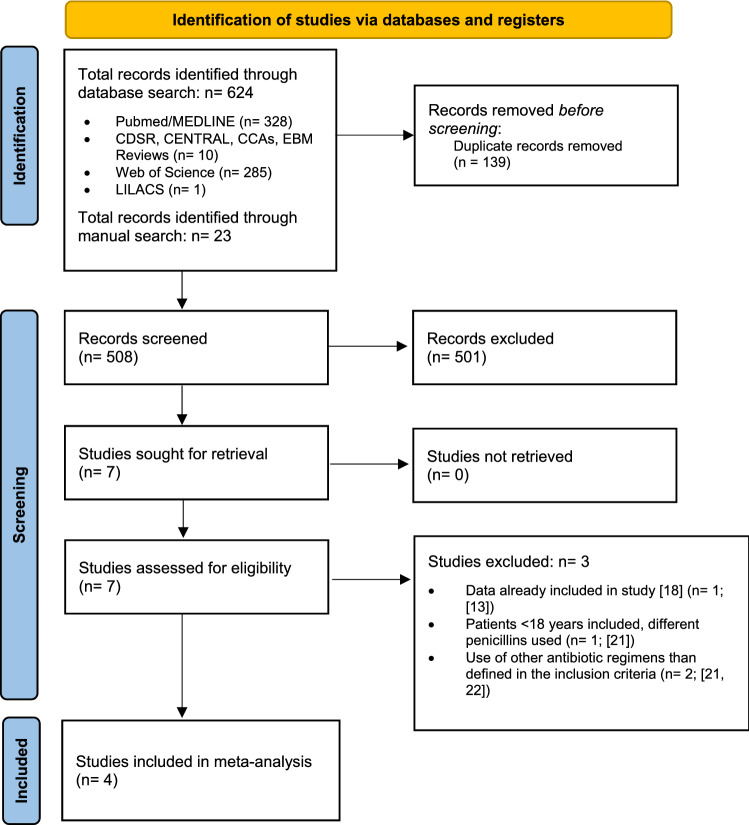
PRISMA flow diagram [15] of literature search and study selection

**Fig. 2:**
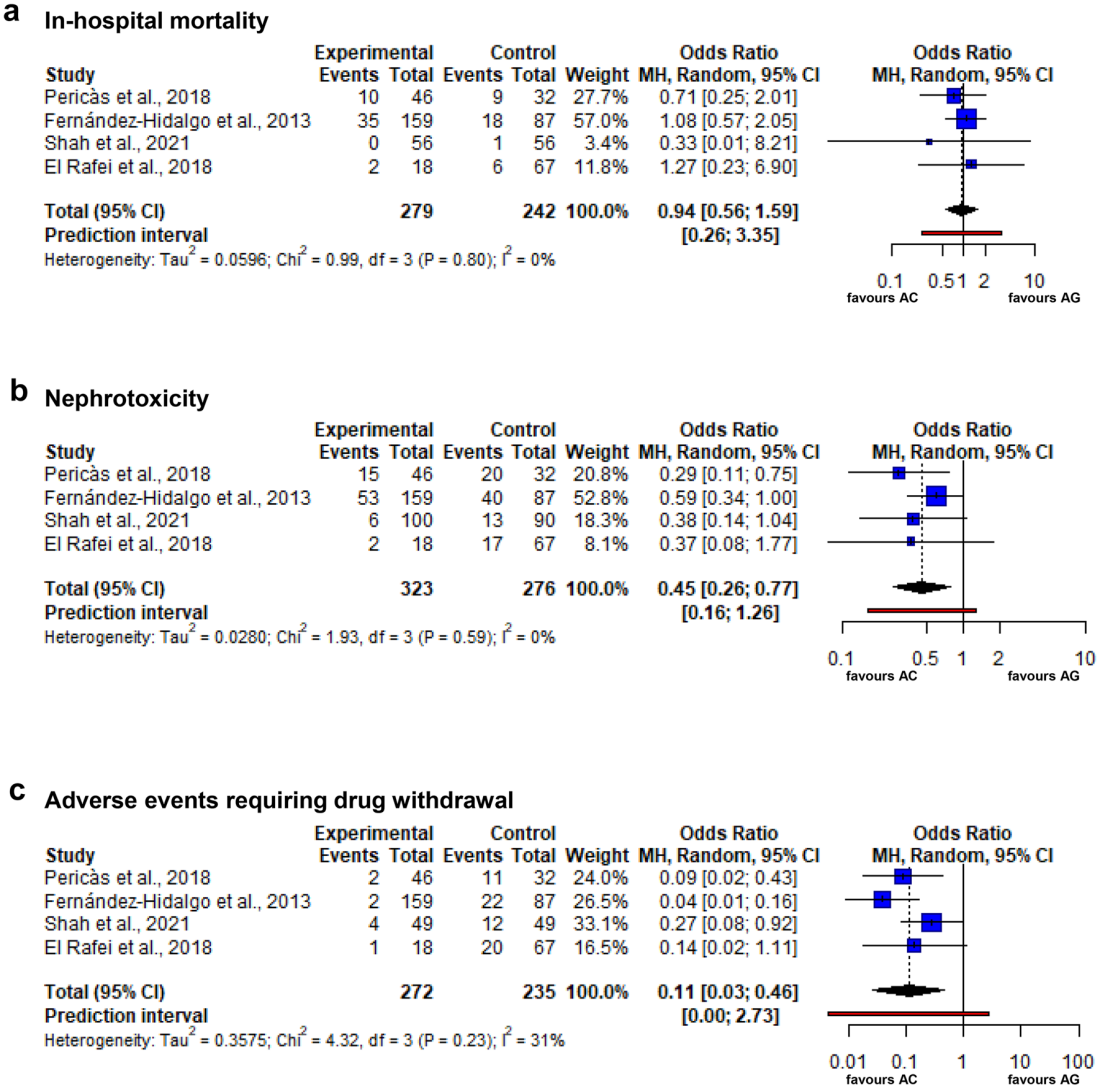
Forest plots of **a** the primary outcome measure ‘in-hospital mortality’, **b** the secondary outcome measure ‘nephrotoxicity’ and **c** the secondary outcome measure ‘adverse events requiring drug withdrawal’. Depicted are the pooled effect estimate (dotted black line), the 95% confidence interval (CI; black diamond) and the prediction interval (red bar)

Figure 1 depicts the PRISMA flow diagram [15] of literature search and study selection of our meta-analysis. After duplicate removal, 508 of the previously identified records were screened, which led to eligibility assessment of 7 studies [12, 13, 18–22]. Of these, 3 studies had to be excluded due to overlapping data (*n* = 1) [13], use of other antibiotic regimens than defined in the inclusion criteria (*n* = 2) [21, 22] or inclusion of patients < 18 years of age (*n* = 1, see Fig. 1) [21]. Characteristics of the 4 included retrospective, nonrandomized, observational cohort studies [12, 18–20] are depicted in Table 1. A summary of the risk of bias assessment is depicted in Supplementary Fig. 1. Of note, we observed no substantial between-study heterogeneity (see Fig. 2a–c and Supplementary Fig. 2).

**Table 1:** Characteristics of the four studies included

Authors/year of publication/reference number	Study design	Patient population	Definition of IE	Drug dosages	Duration of antibiotic treatment	Definition of renal failure	Follow-up
Pericàs et al./2018/[18]	Retrospective, nonrandomized, observational cohort study	Single center, prospective data collection, total *N* = 78	mod. Duke criteria	Ampicillin (2 g/4 h), gentamicin (3 mg/kg/d in 3 doses), ceftriaxone (2 g/12 h)	4–6 weeks	Increase in creatinine of ≥ 0.3 mg/dL or ≥ 50% within 48 h or diuresis ≤ 0.5 mL/kg/h in 6 h	12 months
Fernández-Hidalgo et al./2013/[12]	Retrospective, nonrandomized, observational cohort study	Multicenter (17 hospitals), prospective and retrospective data collection, total *N* = 246	mod. Duke criteria	Ampicillin (2 g/4 h), gentamicin (3 mg/kg/d in up to 3 doses), ceftriaxone (2 g/12 h)	4–6 weeks; median treatment duration 42 days	25% increase of baseline creatinine concentration	3 months
Shah et al./2021/[19]	Retrospective, nonrandomized, observational cohort study; propensity score matching	Multicenter (3 hospitals), retrospective data collection, total *N* = 190	mod. Duke criteria	NA	At least ≥ 48 h; mean treatment duration 42 days, completed in: AC: 78%, AG: 89%	Increase in creatinine of ≥ 0.3 mg/dL or ≥ 50% within 48 h	3 months
El Rafei et al./2018/[20]	Retrospective, nonrandomized, observational cohort study	Single center, retrospective data collection, total *N* = 85	mod. Duke criteria	Ampicillin (2 g/4 h), gentamicin (3 mg/kg/d in 2–3 doses), ceftriaxone (2 g/12 h)	4–6 weeks planned, at least ≥ 48 h; median treatment duration 42 days, completed in: AC: 72%, AG: 51%	RIFLE criteria [33]	12 months

*mod.* modified, *NA* not available

### Dosage and duration of antibiotic therapy

Of the 4 included studies, only 3 [12, 18, 20] reported the exact dosage and duration of antibiotic treatment (see Table 1). These studies investigated patients who received either AC [ampicillin (2 g/4 h) + ceftriaxone (2 g/12 h)] or AG [ampicillin + gentamicin (3 mg/kg/day)] for 4–6 weeks; however, differences were reported in the frequency of gentamicin application (1–3 doses/day).

### In-hospital mortality and 3-month mortality

**Fig. 3:**
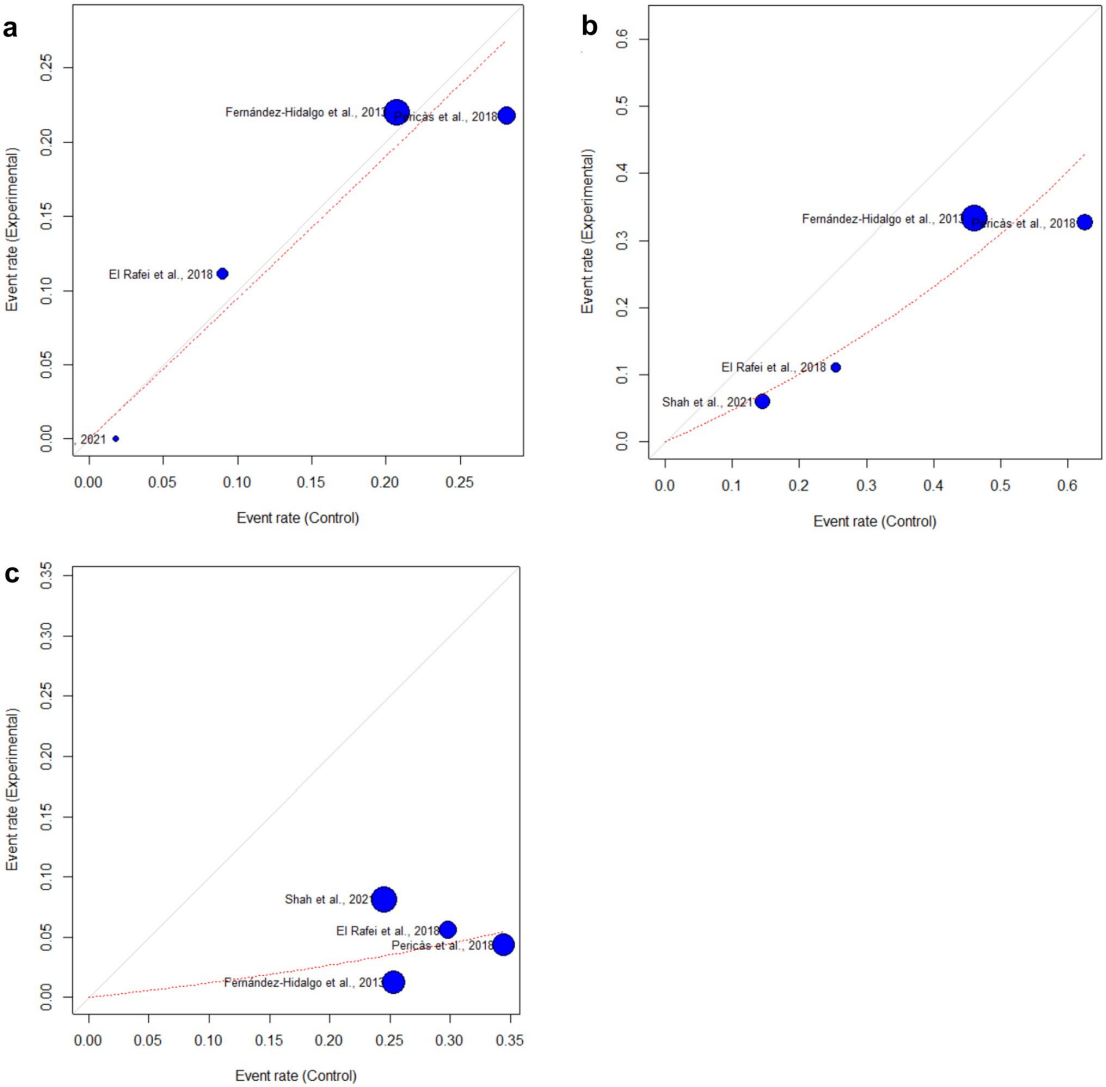
L’Abbé plots of **a** the primary outcome measure ‘in-hospital mortality’, **b** the secondary outcome measure ‘nephrotoxicity’ and **c** the secondary outcome measure ‘adverse events requiring drug withdrawal’. Depicted are event rates of experimental treatment arm (AC) and control treatment arm (AG), as well as the pooled effect estimate (dotted red line)

Data on the primary outcome of in-hospital mortality were available from all 4 studies, whereas data on the secondary outcome of 3-month mortality were available from 3 studies only [12, 19, 20]. There was no statistically significant difference in in-hospital mortality or 3-month mortality between the two treatment arms (in-hospital mortality: OR 0.94 [0.56–1.59], *p* = 0.740, see Figs. 2a and 3a; 3-month mortality: OR 1.31 [0.94–1.84], *p* = 0.072).

### Nephrotoxicity

Data on nephrotoxicity were available from all 4 studies (see Table 1). Of both treatment arms, nephrotoxicity occurred significantly less often in patients receiving AC than in patients treated with AG (OR 0.45 [0.26–0.77], *p* = 0.0182, see Figs. 2b and 3b).

### Adverse events requiring drug withdrawal

Data on drug withdrawal due to adverse events of antibiotic treatment were available from all 4 studies. Drug withdrawal occurred significantly less often in patients treated with AC than in patients treated with AG (OR 0.11 [0.03–0.46], *p* = 0.0160, see Figs. 2c and 3c).

### Relapse within 3 months of follow-up and treatment failure requiring change of antibiotic regimen

Data on relapses were reported in 3 studies [12, 18, 20], whereas data on treatment failure were reported in 2 studies [12, 19]. There were no statistically significant differences in these secondary outcome measures between the two treatment arms investigated (relapses: OR 1.21 [0.07–21.47], *p* = 0.8066); treatment failure: OR 0.47 [0.03–8.16], *p* = 0.1847).

## Discussion

Infective endocarditis (IE) remains one of the most challenging diseases in modern cardiology. With an ever-increasing number of cardiac and extracardiac devices implanted, as well as an increasingly old and multimorbid population, the incidence of endocarditis has been slightly rising in recent years in parts of the world [23–28]. Furthermore, despite the availability of clinical practice guidelines and improvements in therapy itself, mortality of IE remains high with an estimated 12-month mortality rate of 15–30% reported in most studies [3, 27, 29]. Besides morbidity and mortality exerted by the infection itself, the intensive and tedious antibiotic treatment regimens of IE are in part associated with an increased risk for adverse outcomes, such as drug-induced nephrotoxicity or ototoxicity in patients treated with aminoglycosides, rendering endocarditis and its necessary therapies a potentially debilitating disease entity for affected patients [30].

Currently, guidelines by the European Society of Cardiology (ESC) recommend either a combination of ampicillin plus ceftriaxone (AC) or amoxicillin/ampicillin plus gentamicin (AG) in the treatment of *Enterococcus faecalis* infective IE [3]. However, among the aforementioned antibiotic drugs, gentamicin is associated with the highest risk of drug-induced adverse events, including potentially irreversible complications such as vestibulotoxicity or ototoxicity [7, 8]. As guidelines currently state an equivalent class IB recommendation for both antibiotic regimens [3], the aim of this meta-analysis was to investigate the therapeutic non-inferiority of AC versus AG, and to elucidate whether AC would be associated with a lower risk of drug-induced adverse events.

After including four retrospective, nonrandomized, observational cohort studies [12, 18–20] in this meta-analysis, we found that the AC regime was indeed non-inferior to AG in *E. faecalis* endocarditis, as depicted by no statistically significant differences in hospital mortality (*p* = 0.740), 3-month mortality (*p* = 0.072), relapses (*p* = 0.8066) or treatment failure (*p* = 0.1847) between the two treatment arms investigated. Furthermore, the presumed lower toxicity of the AC regime was confirmed by a significantly lower prevalence of nephrotoxicity (OR 0.45 [0.26–0.77], *p* = 0.0182) and drug withdrawal due to adverse events (OR 0.11 [0.03–0.46], *p* = 0.0160) when compared to patients receiving AG.

Our findings, showing non-inferiority of the AC regime, as well as a significantly reduced risk of drug-induced adverse events, give rise to speculation that AC might be favourable over AG for affected patients with *E. faecalis* endocarditis. Especially in highlight of an increasingly old and multimorbid population, with a steadily incrementing prevalence of chronic kidney disease [31], the aforementioned findings should be taken into consideration when clinical decisions on antibiotic treatment in enterococcal IE are to be made. Moreover, the fact that high-level aminoglycoside-resistant (HLAR) strains of *Enterococci* increasingly limit the use of AG in IE [6, 32] further strengthens the notion to abandon AG in favour of AC in this disease entity.

However, while exhibiting a good tolerability profile [33] and broad antimicrobial spectrum, treatment with ceftriaxone was previously identified as a major risk factor for *Clostridium difficile* infection, as well as for colonization with vancomycin-resistant *E. faecium* (VRE) or other multi-resistant bacteria [34–37]. The link to colonization with these pathogens*,* and its potential implications for the individual patient, as well as the patient’s environment, should be considered when therapeutic decisions on antimicrobial treatment in IE are to be made.

Furthermore, both AC and AG are currently implemented as therapeutic regimens administered via repeated intravenous infusions. However, the Partial Oral Treatment of Endocarditis (POET) trial [38] recently found that a change to oral antibiotics after an initial intravenous phase was non-inferior to continued intravenous treatment in patients with IE. Since a reduction of hospital stay diminishes the risk of complications, associated costs, as well as psychological distress for our patients [38], outpatient treatment appears to be a promising opportunity in patients with *E. faecalis* IE as well. However, treatment with cephalosporins was not investigated in the POET trial, and bioavailability of these antibiotic drugs is unfortunately comparatively low [39]. Nonetheless, novel formulations are being investigated at the moment, which could help to put outpatient treatment with AC in patients with *E. faecalis* IE into practice in the near future [40, 41].

Finally, other antibiotic regimens are also depicted in the ESC guidelines on enterococcal IE, and can thus also be considered in affected patients [3]. However, vancomycin with gentamicin has a weaker class IC recommendation than AC or AG, and treatment with daptomycin is currently recommended in patients with strains resistant to aminoglycosides, beta-lactams and vancomycin only. Furthermore, a recent study by Cerón et al. showed worse microbiological and clinical responses in patients treated with daptomycin when compared to standard treatment [22].

In conclusion, treatment with AC was non-inferior to treatment with AG, and it was associated with a reduced prevalence of nephrotoxicity and drug withdrawal due to adverse events. Thus, combination therapy with AC appears favourable over AG in patients with *E. faecalis* IE.

## Conclusions

Treatment with ampicillin plus ceftriaxone (AC) was non-inferior to ampicillin plus gentamicin (AG) in *E. faecalis* endocarditis, as depicted by no statistically significant differences in-hospital mortality, 3-month mortality, relapses or treatment failure. Furthermore, AC was associated with a lower prevalence of nephrotoxicity and drug withdrawal due to adverse than AG. Given that it was non-inferior and associated with a reduced risk for drug-induced adverse events, AC might thus be favourable over AG in patients with *E. faecalis* IE.

## Limitations

Our study has several limitations. First, all four included studies were relatively small, resulting in a total *N* of only 599 patients included in the current meta-analysis. The low number of included patients, as well as the potential statistical implications thereof, has to be considered when interpreting the results of our study. Moreover, the definition of acute renal failure was slightly different between included studies (also see Table 1), which is why a certain bias on the secondary outcome measure ‘nephrotoxicity’ cannot be excluded. Of note, we only included data of patients with creatinine increase ≥ 50% from the study by El Rafei et al. [20, 42] to meet the definitions of the other included studies. In one study, the definition of acute renal failure was initially missing [19]; however, the corresponding author could provide the definition used when contacted.

Also, between-study heterogeneity was assessed by calculating *τ*^*2*^, *I*^*2*^, *Q* and prediction intervals. Here, we found no substantial heterogeneity; however, a low number of included studies (*k* = 4) can result in a bias of heterogeneity measures [43, 44], which is a known problem of small meta-analyses. Hence, a certain degree of between-study heterogeneity cannot certainly be excluded (see also Supplementary Fig. 2). Also, a low *k* is a problem when assessing publication bias, especially when small study effects should be calculated. Hence, a certain degree of publication bias can also not certainly be excluded. However, for visual estimation, contour-enhanced funnel plots are additionally depicted in Supplementary Fig. 3. The possible biases arising from a low number of included studies should be considered when interpreting the findings of our meta-analysis.

## Supplementary Information

Below is the link to the electronic supplementary material.

**Suppl. Fig. 1:** Summary of the risk of bias assessment. Colors depict the risk of bias in the respective category: red= ‘High’, orange= ‘Unclear’, green= ‘Low’. Supplementary file1 (PPTX 217 kb)

**Suppl. Fig. 2:** Baujat plots of the various outcome measures (1= [18], 2= [12], 3= [19], 4= [20]): **a)** in-hospital mortality, **b)** 3-month mortality, **c)** nephrotoxicity, **d)** adverse events requiring drug withdrawal, **e)** relapses, **f)** treatment failure. Depicted are squared Pearson residuals of the studies (x-axis; corresponds to contribution to the *Q*-test statistic for heterogeneity) and the influence on the overall result (y-axis). Supplementary file2 (PPTX 266 kb)

**Suppl. Fig. 3:** Contour-enhanced funnel plots of the various outcome measures: **a)** in-hospital mortality, **b)** 3-month mortality, **c)** nephrotoxicity, **d)** adverse events requiring drug withdrawal, **e)** relapses, **f)** treatment failure. Supplementary file3 (PPTX 499 kb)

## Data Availability

The data underlying this study will be shared upon reasonable request to the corresponding author.
