# Supplementary figures and images for: Time to abandon ampicillin plus gentamicin in favour of ampicillin plus ceftriaxone in Enterococcus faecalis infective endocarditis? A meta-analysis of comparative trials
Source: Clin Res Cardiol. 2021 Nov 9;111(10):1077–86. doi: 10.1007/s00392-021-01971-3 (PMC9525249; doi:10.1007/s00392-021-01971-3)

## Slide 1
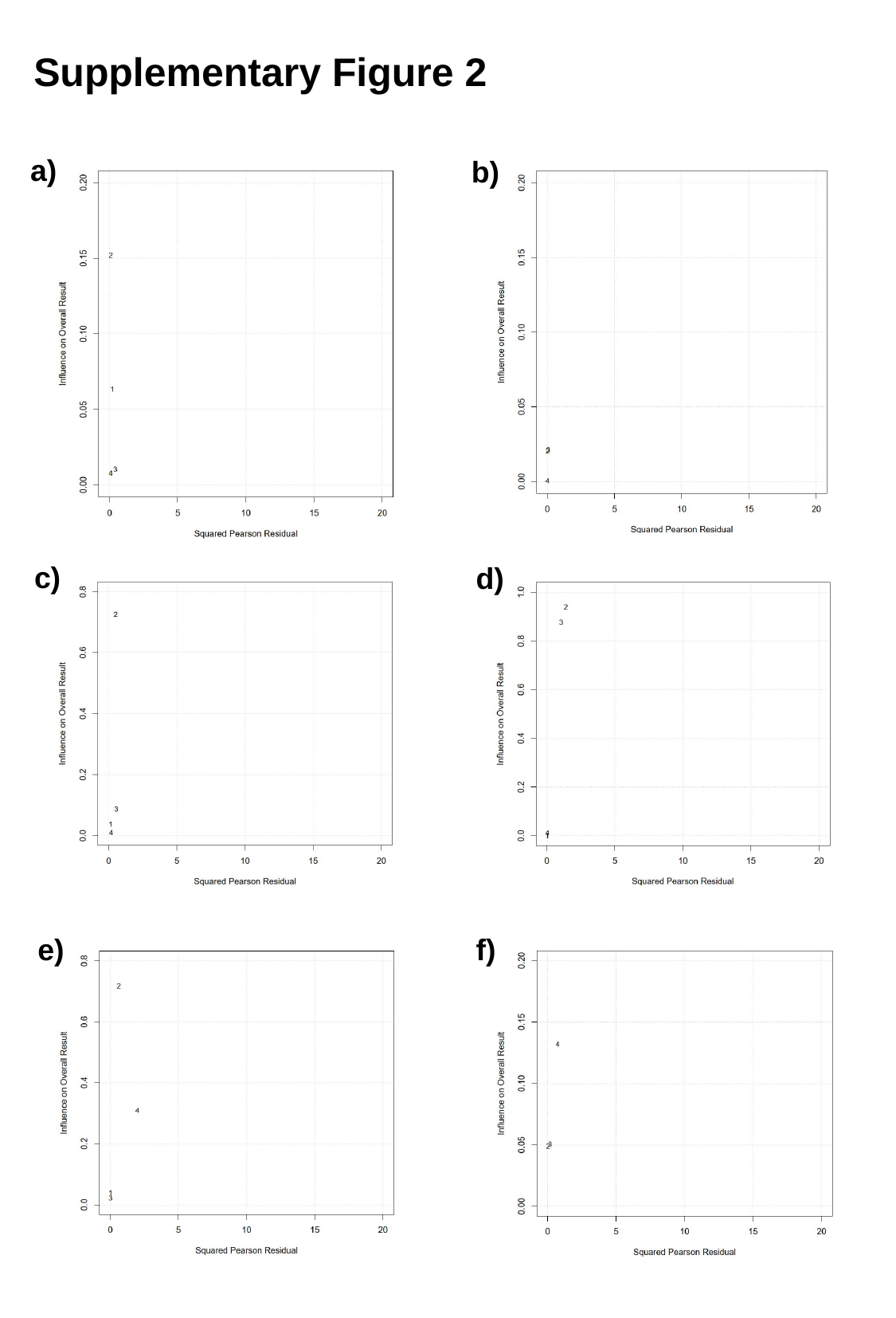

Supplementary Figure 2
a)
b)
c)
d)
e)
f)

Supplement: Supplementary file 1 — Suppl. Fig. 1: Summary of the risk of bias assessment. Colors depict the risk of bias in the respective category: red= ‘High’, orange= ‘Unclear’, green= ‘Low’. Supplementary file1 (PPTX 217 kb) [file 392_2021_1971_MOESM1_ESM.pptx]

## Slide 1
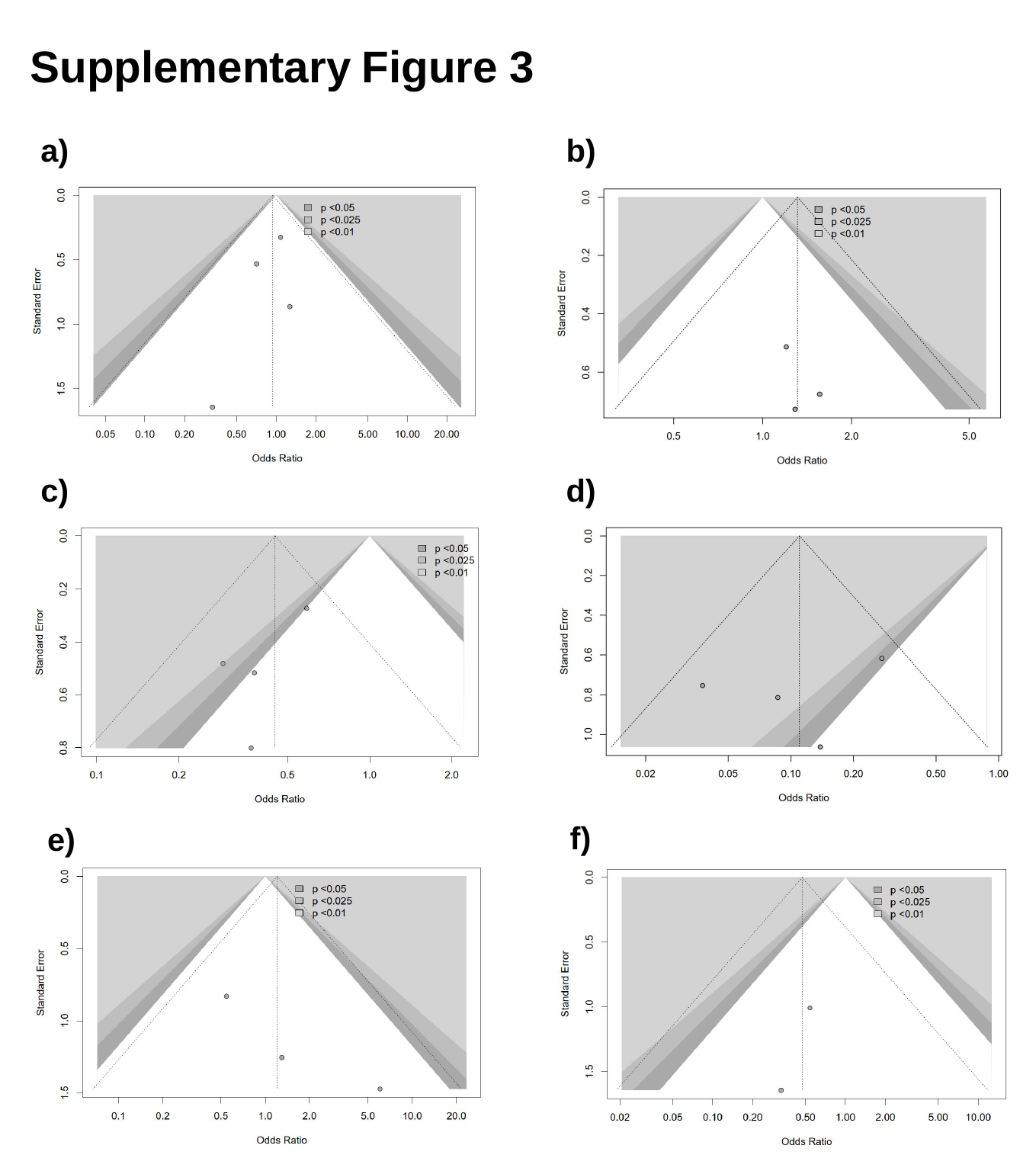

Supplementary Figure 3
a)
b)
c)
d)
f)
e)

Supplement: Supplementary file 2 — Suppl. Fig. 2: Baujat plots of the various outcome measures (1= [18], 2= [12], 3= [19], 4= [20]): a) in-hospital mortality, b) 3-month mortality, c) nephrotoxicity, d) adverse events requiring drug withdrawal, e) relapses, f) treatment failure. Depicted are squared Pearson residuals of the studies (x-axis; corresponds to contribution to the Q-test statistic for heterogeneity) and the influence on the overall result (y-axis). Supplementary file2 (PPTX 266 kb) [file 392_2021_1971_MOESM2_ESM.pptx]

## Slide 1
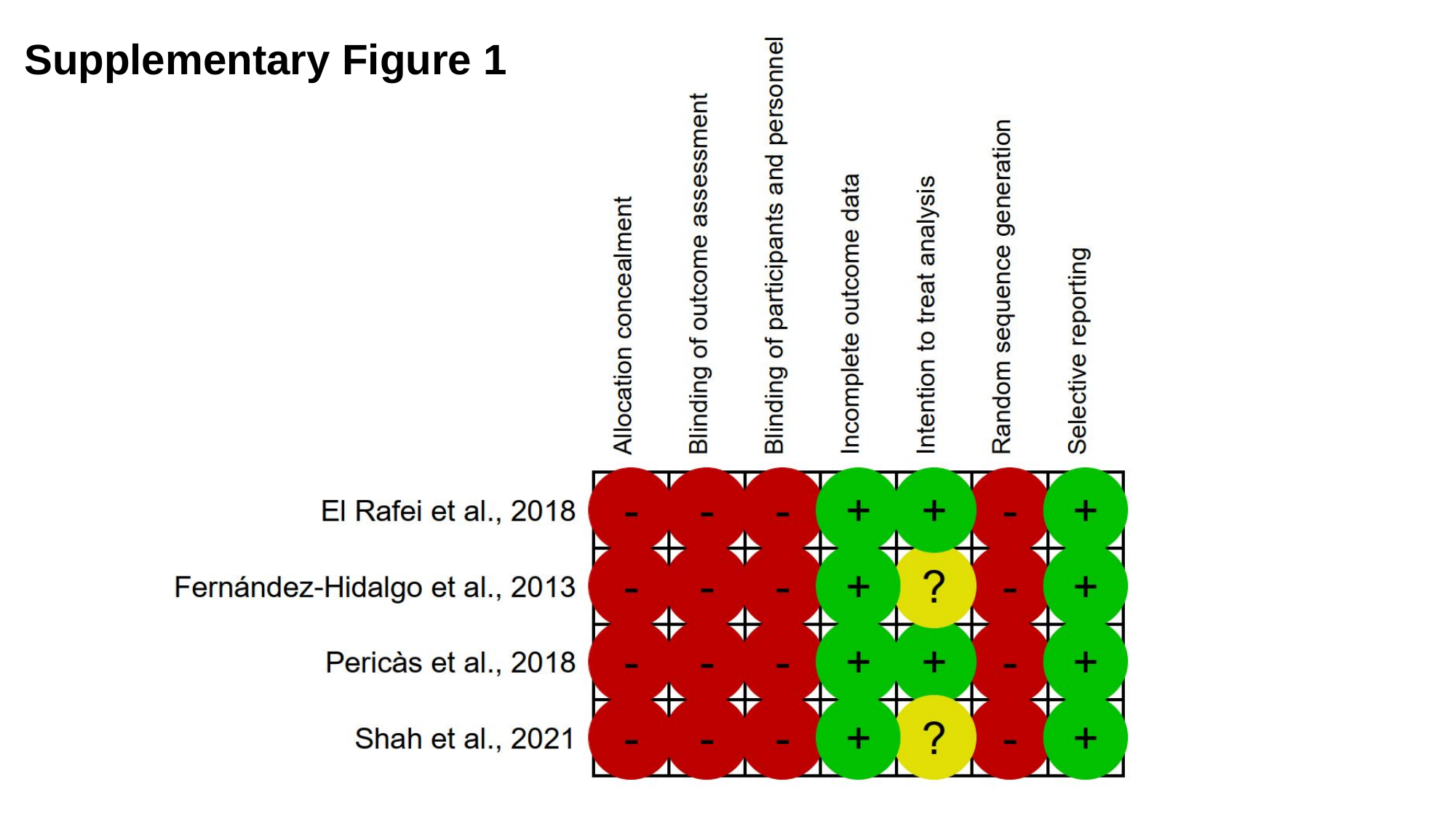

# Supplementary Figure 1

Supplement: Supplementary file 3 — Suppl. Fig. 3: Contour-enhanced funnel plots of the various outcome measures: a) in-hospital mortality, b) 3-month mortality, c) nephrotoxicity, d) adverse events requiring drug withdrawal, e) relapses, f) treatment failure. Supplementary file3 (PPTX 499 kb) [file 392_2021_1971_MOESM3_ESM.pptx]
